# The Effects of Chronic Ankle Instability on the Biomechanics of the Uninjured, Contralateral Ankle During Gait

**DOI:** 10.1111/os.13307

**Published:** 2022-07-19

**Authors:** Elaheh Ziaei Ziabari, Mohammad Haghpanahi, Mohammad Razi, Bart Lubberts, Soheil Ashkani‐Esfahani, Christopher W. DiGiovanni

**Affiliations:** ^1^ School of Mechanical Engineering Iran University of Science and Technology Tehran Iran; ^2^ Foot & Ankle Research and Innovation Laboratory (FARIL), Department of Orthopaedic Surgery, Massachusetts General Hospital Harvard Medical School Boston Massachusetts USA; ^3^ Department of Orthopedic Surgery, Rasoul Akram Hospital Iran University of Medical Sciences Tehran Iran; ^4^ Foot & Ankle Service, Massachusetts General Hospital, Newton‐Wellesley Hospital Harvard Medical School Massachusetts General Hospital, Boston Massachusetts USA

**Keywords:** ankle sprain, chronic ankle instability, biomechanics, kinematics, gait

## Abstract

**Objective:**

To determine whether unilateral chronic ankle instability (CAI) affects the kinematics of the uninjured contralateral ankle.

**Methods:**

In this case‐control study, 15 adult patients with unilateral CAI and 15 healthy controls were studied. Both the unstable and uninjured ankles in patients with unilateral CAI (CAI group, *n* = 15) were compared with that of healthy individuals (control group, *n* = 15). Applying body photo‐reflective markers, the participant's motion during gait was measured. Biomechanical variables including overall ankle‐toe angle, linear velocity, linear acceleration, angular velocity, angular acceleration, range of motion (RoM) in dorsiplantar flexion, and inversion‐eversion at initial contact, loading response, mid‐stance, terminal stance, pre‐swing, and swing phase of the gait were measured.

**Results:**

In patients with CAI, the injured and uninjured ankles were significantly different regarding angle‐toe angle, inversion‐eversion RoM, dorsiplantar flexion in mid‐stance, inversion‐eversion at initial contact and terminal stance as well as the pre‐swing and swing phases (*p* < 0.01). The uninjured ankles of patients showed lower ankle‐toe velocity (*p* = 0.01) and acceleration (*p* = 0.01) compared to both the left and right ankles of the controls. In addition, the uninjured ankles of the patients showed decreased ankle dorsiflexion and increased inversion during initial contact, loading response, mid‐stance, terminal stance, pre‐swing, and swing compared to the control group (*p* < 0.017).

**Conclusion:**

The results suggest that unilateral CAI can affect gait biomechanics in the contralateral uninjured ankle. Left unaddressed, unilateral CAI may lead to increased morbidity to the contralateral uninjured side. When surgery is not preferred for the management of unilateral CAI, rehabilitation protocols should focus on both sides.

## Graphical abstract

- Unilateral chronic lateral ankle instability affects gait biomechanics in the contralateral uninjured ankle.
- Mismanaged unilateral chronic lateral ankle instability may increase the risk of progressive disability to the contralateral uninjured side.
- When surgery is not preferred to manage chronic lateral ankle instability, rehabilitation protocols should focus on both sides.

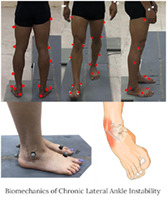

Unilateral chronic ankle instability affects the contralateral healthy ankle

## Introduction

Ankle sprains are among the most prevalent injuries. A high percentage of individuals with ankle sprains will develop residual physical disability which may include chronic ankle instability (CAI). Accordingly, an estimated 15%–20% of the patients with acute ankle sprains may develop chronic lateral ankle instability CAI.^1^

Treatment of CAI includes conservative treatment or surgery that focuses mainly on the injured ankle with instability. Surgical treatment for these patients is usually considered after conservative treatment has failed to restore stability.^2^

Previous biomechanical research on patients diagnosed with CAI showed considerable changes in the kinematics and kinetics of the unstable ankle when compared to uninjured ankles in healthy individuals during gait.^3^, ^4^, ^5^, ^6^ Without adequate treatment, these defects may lead to long‐term morbidities and degenerative changes of the ankle joint over time^7^, ^8^, ^9^ Previous literature has described that CAI resulted in a significantly greater inversion in the frontal plane and a higher angular velocity at heel strike.^9^ A higher inversion was observed in all walking speeds in the affected ankle in these studies.^8^ While healthy ankles were controlled by an invertor muscle moment working eccentrically during the early stance phase of the gait, CAI has led to the utilization of an evertor muscle moment working concentrically.^9^ These alterations in the kinematics were assumed to result in increased stress on the joint during the heel strike, increased loading response phases of the gait, and repeated injury to the ankle joint structures.^8^, ^9^

In a prospective study by Doherty *et al*., patients with CAI showed inability to complete jumping and landing within 2 weeks and poorer dynamic postural control 6 months after a first‐time lateral ankle sprain. They showed that the single‐leg drop and drop vertical jump were important predictive inputs for CAI outcomes including dynamic postural control and patient self‐reported functions after 6 months.^10^

Chinne and colleagues compared the ankle joint among patients with CAI and healthy controls. In this study, the authors showed that those with CAI had altered kinematics at specific points of the gait cycle.^11^ Changes in the kinematics of the ankle joint of individuals with CAI were also seen in the study by Delahunt and colleagues where they compared the ankles of healthy individuals with that of patients with CAI.^12^ As expected and as these studies have shown, biomechanical differences were detected in ankles with instability compared to healthy ankles.^12^ These changes in the biomechanics of the ankle joint after CAI have also been documented in other studies focusing on the kinetic and kinematic changes within the ankle joint. However, there has been an ongoing debate, as to whether or not unilateral CAI can lead to biomechanical dysfunction and injuries of the same individual's contralateral, uninjured side.^13^, ^14^

We hypothesized that the injured ankle in patients with CAI will affect the biomechanics of the contralateral uninjured ankle of these patients. The objectives of this in‐vivo biomechanical study were (i) to assess bilateral ankle kinematics in patients diagnosed with unilateral CAI, and (ii) to compare these findings with those found in healthy individuals. Accordingly, in this study, we compared the kinematics of the injured ankle joint of patients with CAI with that of healthy controls and their own contralateral uninjured ankles.

## Methods and Materials

### Study Design

The protocol of this case‐control study was approved by the Institutional Review Board of the Iran University of Science and Technology, Tehran, Iran (Registry no.: 2580373). Study participants were enrolled in three orthopaedic surgical clinics in Tehran, Iran. The protocol was explained to each participant and informed consent was obtained before entering the study. Inclusion criteria were: (i) individuals between 18 and 65 years old age; and (ii) suffering from unilateral CAI. The exclusion criteria were individuals with: (i) musculoskeletal disorders other than injuries of the lateral ligaments of the ankle complex, such as joint hyperlaxity, syndesmosis ligament injury, or deltoid ligament injury; (ii) neurological disorders such as neuropathies and lower limb paralysis; (iii) signs of pain or swelling of the lower extremity due to other injuries and not merely related to CAI; (iv) psychiatric disorders preventing appropriate cooperation; and (v) individuals who did not provide informed consent.

A total of 15 individuals diagnosed with unilateral functional CAI were included in the patient group between November 2018 and October 2019. The diagnosis of CAI was made if patients had clinical symptoms of: instability, including giving way for more than 6 months on multiple occasions; a history of at least one ankle sprain within the last 12 months before referral; signs of lateral ankle ligaments injury in anterior drawer and talar tilt tests; radiographic and MRI images based on previously reported criteria.^15^, ^16^, ^17^ The contralateral ankles of the patients were also evaluated and examined in order to rule out the presence of any instability and signs and symptoms of previous injuries. After physical examination by an expert orthopaedic surgeon and magnetic resonance imaging (MRI) to confirm lateral ligament injury,^16^ individuals were referred to a biomechanics laboratory. The control group consisted of 15 randomly selected healthy individuals. These study participants had no abnormalities of the foot and ankle during the physical examination and had no history of ankle sprain or fracture.

### Three‐Dimensional Motion and Gait Analysis

**Fig. 1:**
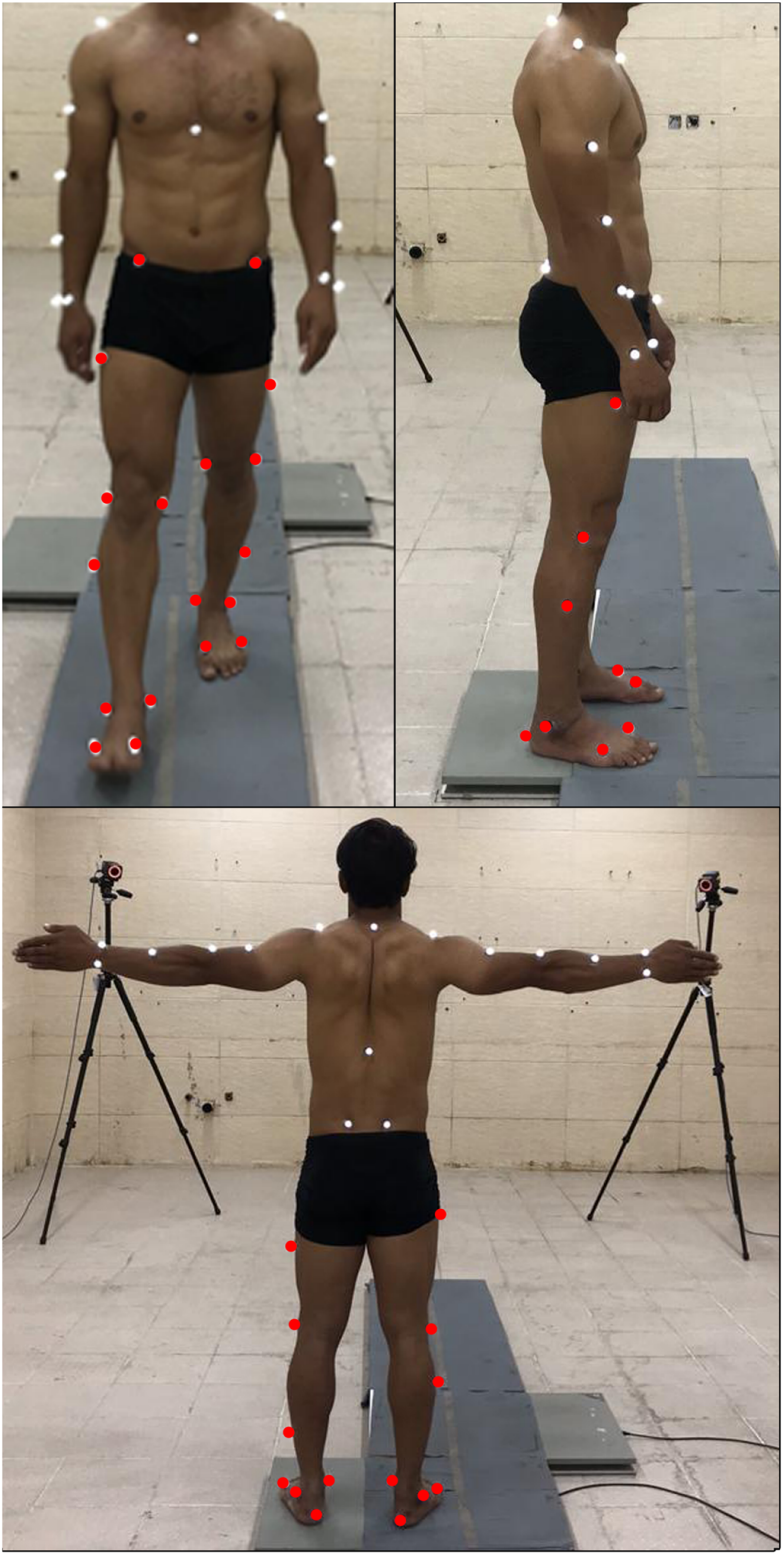
Locations of the markers on the body in the Plug‐in Gait model. Red marks show the locations of the markers on the lower limbs

Anthropometric indices including height, weight, and body mass index (BMI) were measured. According to the Vicon™ Plug‐in‐Gait model (Vicon, Oxford, UK),^18^ 18 body markers were placed on each study participant in order to measure motion during gait (Table 1, Figure 1). For adequate measurement of kinematic variables, patients were asked to walk barefoot with minimal clothing through a walkthrough path and to walk regularly as their routine habit. Three‐dimensional kinematic parameters were measured using six Vicon cameras (120 Hz) and the Nexus software (version 2.5; Sonatype™, Fulton, USA) that monitored the individual's motion pattern during the gait. All devices were calibrated before use. The settings of the motion analyzer and the force plate were organized in a way that the anterior‐posterior directions were in accordance with the "Y" axis, the medial‐lateral directions were in accordance with the "X" axis, and the vertical directions were following the "Z" axis. Angles of dorsi‐ or plantarflexion (the total sagittal plane arch of RoM of the ankle) and inversion and eversion (the total coronal plane arch of RoM of the ankle) were measured at different gait stages.

**TABLE 1:** Location of each marker on the lower limb in the Plug‐in Gait model

No.	Right	No.	Left
1	Posterior inferior iliac spine	2	Posterior inferior iliac spine
3	Anterior superior iliac spine	4	Anterior superior iliac spine
5	Thigh	6	Thigh
7	Knee	8	Knee
9	Leg	10	Leg
11	Ankle	12	Ankle
13	Heel	14	Heel
15	Toe	16	Toe
17	Metatarsus 5	18	Metatarsus 5

Overall ankle‐toe linear speed (m/s), overall ankle‐toe linear acceleration (m/s^2^), leg absolute angle (the angle between the toe and the leg considering the horizontal plane as base zero), angular velocities of the ankle‐toe (m/s), the angular acceleration of the ankle‐toe (m/s^2^), range of motion (RoM) in dorsi‐plantar flexion (the total sagittal plane RoM arch of the ankle), and degree of eversion and inversion (the total coronal plane arch of RoM of the ankle) were measured during the gait. Initially, all data were calculated in the X, Y, and Z axes. Data were extracted and classified using MatLab 7.04 (Mathworks Inc., Natick, MA).

### Statistical Analysis

Data obtained from three‐axis measurements were analyzed using the SPSS version 21.0 (IBM Inc., Chicago, IL, USA) and were reported as mean ± standard deviation (SD). One‐way ANOVA and Tukey's post hoc tests were used to test the hypotheses that unilateral CAI affects the kinematics of the healthy contralateral ankle during gait. Considering dorsiplantar flexion as our specific variable, as the effect size was a large number (14.4, Mean Difference = 7.2, SD = 0.5, based on Wilcoxon signed‐rank test), the minimum sample size with respect to a power of 95% and type one error of 1% was estimated to be lower than 1. Decreasing the effect size to 2 and 3 resulted in a minimum sample size of 9 and 4, respectively. Thus, based on a previous publication,^11^ we decided to consider a larger sample size of 15 participants in each group in order to validate our outcome. In this study, the following comparisons in ankle kinematics were conducted: unstable *vs* stable contralateral side in patients; stable ankle in the patient group *vs* each side of the control group; and left *vs* right ankles in the control group. A Bonferroni correction was applied to correct for multiple comparisons. A *p*‐value of less than 0.0167 (0.05/3) was considered statistically significant.

## Results

### General Results

The CAI group (*n* = 15) consisted of eight males and seven females, with a mean age of 29.7 ± 10.5 years (range: 18–50 years) and BMI of 22.8 ± 6.9 kg/m^2^. The control group included nine males and six females with a mean age of 31.06 ± 7.31 years (range: 18–65 years) and BMI of 23.3 ± 2.8 kg/m^2^).

#### Comparison of the bilateral ankles in each group

A comparison of right and left ankles in the control group showed no significant differences with respect to any measured gait parameter. These included ankle‐toe degrees, ankle‐toe velocity, ankle‐toe acceleration, ankle‐toe angular velocity, ankle‐toe angular acceleration, dorsoplantar flexion RoM, and inversion‐eversion RoM (Table 2). A comparison between the unstable and stable ankles in the CAI group demonstrated several distinct and significant differences in biomechanical responses (Table 3). More specifically, gait kinematics analysis showed substantial differences between ankles in the study population. There were significant decreases in the ankle‐toe angle (−4.4° ± 17.8° *vs* 17.9° ± 6.1°; *p* < 0.001) as well as significant increases in inversion‐eversion RoM (19.6° ± 9.8° *vs* 12.9° ± 7.3°; *p* = 0.002). Dorsiflexion position of the joint in unstable ankles decreased significantly during the midstance phase of the gait (8.8° ± 3.9° *vs* 11.2° ± 5.9°; *p* = 0.004). Inversion‐eversion positioning of the ankle decreased significantly during initial contact (8.4° ± 4.1° *vs* 13.5° ± 3.8°; *p* = 0.002) and terminal stance (7.5° ± 4.7° *vs* 11.5° ± 3.2°; *p* = 0.004) but increased significantly during pre‐swing (−3.6° ± 1.9° *vs* −5.9° ± 3.8°; *p* = 0.004) and swing phases (13.8° ± 4.4° *vs* 10.8° ± 4.9°; *p* = 0.002; Table 3).

**TABLE 2:** Comparison of kinematic measurements during the gait, degrees of dorsiplantar flexion, and inversion‐eversion in different phases of the gait, between the two uninjured ankles in the control group

Variables	Control group	Control group	p‐value*
	Right Ankle (*n* = 15) (mean ± SD)	Left Ankle (*n* = 15) (mean ± SD)	
Ankle‐toe (degrees)	18.7 ± 4.1	18.4 ± 3.7	0.21
Ankle‐toe velocity (m/s)	1.1 ± 0.1	1.2 ± 0.2	0.16
Ankle‐toe acceleration (m/s^2^)	8.5 ± 1.6	9.9 ± 2.8	0.45
Ankle‐toe angular velocity (radiant/s)	0.5 ± 0.1	0.3 ± 0.1	0.41
Ankle‐toe angular acceleration (radiant/s^2^)	1.5 ± 0.4	1.8 ± 0.3	0.47
Dorsoplantar flexion RoM (degrees)	93.7 ± 3.9	90.5 ± 3.0	0.75
Inversion‐eversion RoM (degrees)	26.9 ± 4.6	25.6 ± 2.4	0.92
Dorsiplantar flexion^a^			
Initial contact	6.3 ± 1.4	5.1 ± 1.5	0.92
Loading response	0.5 ± 1.4	−0.3 ± 1.2	0.74
Mid‐stance	17.8 ± 2.5	14.7 ± 3.9	0.41
Terminal stance	−4.5 ± 1.9	−6.7 ± 4.9	0.16
Pre‐swing	−8.8 ± 3.9	−10.4 ± 3.9	0.46
Swing	8.4 ± 4.7	8.8 ± 3.4	0.51
Inversion‐Eversion^b^			
Initial contact	1.5 ± 3.8	1.9 ± 5.2	0.43
Loading response	−3.4 ± 4.1	−4.1 ± 2.5	0.09
Mid‐stance	6.3 ± 3.6	4.6 ± 3.3	0.11
Terminal stance	3.9 ± 3.4	4.4 ± 5.6	0.13
Pre‐swing	−9.6 ± 4.8	−11.3 ± 3.9	0.27
Swing	4.7 ± 4.1	6.2 ± 5.1	0.37

Abbreviations: RoM, Range of Motion

a Positive values show plantar flexion and negative values indicate dorsiflexion

^b^ Positive values show inversion and negative values indicate eversion.

*p < 0.017 was considered as statistically significant.

**TABLE 3:** Comparison of kinematic measurements during the gait between the *unstable* ankle and the *stable* contralateral ankle in patients diagnosed with unilateral chronic ankle instability

Variables	Patient group	Patient group	p‐value*
	Unstable Ankle (*n* = 15) (mean ± SD)	Stable Ankle (*n* = 15) (mean ± SD)	
Ankle‐toe (degrees)	−4.4 ± 17.8	17.9 ± 6.1	**<0.001**
Ankle‐toe velocity (m/s)	0.6 ± 0.1	0.7 ± 0.2	0.97
Ankle‐toe acceleration (m/s^2^)	5.9 ± 1.6	6.3 ± 1.5	0.38
Ankle‐toe angular velocity (radiant/s)	0.3 ± 0.2	0.4 ± 0.2	0.36
Ankle‐toe angular acceleration (radiant/s^2^)	1.5 ± 0.7	1.7 ± 1.4	0.74
Dorsoplantar flexion RoM (degrees)	92.2 ± 3.2	93.2 ± 3.5	0.18
Inversion‐eversion RoM (degrees)	19.6 ± 9.8	12.9 ± 7.3	**0.002**
Dorsiplantar flexion^a^			
Initial contact	−3.9 ± 1.9	−5.0 ± 2.7	0.21
Loading response	−4.2 ± 1.9	−5.8 ± 0.9	0.14
Mid‐stance	8.8 ± 3.9	11.2 ± 5.9	**0.004**
Terminal stance	−0.9 ± 2.4	−1.2 ± 1.1	0.14
Pre‐swing	−15.1 ± 3.8	−13.1 ± 2.6	0.09
Swing	5.8 ± 2.5	4.8 ± 6.2	0.19
Inversion‐Eversion^b^			
Initial contact	8.4 ± 4.1	13.5 ± 3.8	**0.002**
Loading response	3.4 ± 1.7	4.4 ± 3.2	0.37
Mid‐stance	13.7 ± 5.1	12.9 ± 7.4	0.78
Terminal stance	7.5 ± 4.7	11.5 ± 3.2	**0.004**
Pre‐swing	−3.6 ± 1.9	−5.9 ± 3.8	**0.004**
Swing	13.8 ± 4.4	10.8 ± 4.9	**0.002**

Bold indicates significance values *p* < 0.05.

Abbreviations: RoM, Range of Motion

a Positive values show plantar flexion and negative values indicate dorsiflexion

^b^ Positive values show inversion and negative values indicate eversion.

*p= 0.017 was considered as statistically significant.

#### Comparison of ankle biomechanics between patients and controls

When comparing the uninjured side of the CAI group with the right and left ankles of the control population, ankle‐toe velocity (0.7 ± 0.2 *vs* 1.1 ± 0.1; *p* = 0.01 and 0.7 ± 0.2 *vs* 1.2 ± 0.2; *p* = 0.01, respectively) and acceleration of movement during gait (6.3 ± 1.5 *vs* 8.5 ± 1.6; *p* = 0.01 and 6.3 ± 1.5 *vs* 9.9 ± 2.8; *p* = 0.01, respectively) were found to be significantly decreased in the CAI group (Table 4). This started to appear at 50% and 58% of the gait cycle and resolved at 98% of the gait cycle.

**TABLE 4:** Comparison of kinematic measurements during the gait, degrees of dorsiplantar flexion, and inversion‐eversion in different phases of the gait, between the stable ankle in the CAI group and the stable ankles in the control group

Variables	CAI group Stable Ankle (*n* = 15) (mean ± SD)	Control group Right Ankle (*n* = 15) (mean ± SD)	*p‐value*	Control group Left Ankle (*n* = 15) (mean ± SD)	*p‐value*
Ankle‐toe (degrees)	17.9 ± 6.1	18.7 ± 4.1	0.84	18.4 ± 3.7	0.82
Ankle‐toe velocity (m/s)	0.7 ± 0.2	1.1 ± 0.1	**0.01**	1.2 ± 0.2	**0.01**
Ankle‐toe acceleration (m/s^2^)	6.3 ± 1.5	8.5 ± 1.6	**0.01**	9.9 ± 2.8	**0.01**
Ankle‐toe angular velocity (radiant/s)	0.4 ± 0.2	0.5 ± 0.2	0.43	0.3 ± 0.1	0.69
Ankle‐toe angular acceleration (radiant/s^2^)	1.7 ± 1.4	1.5 ± 0.4	0.46	1.8 ± 0.3	0.53
Dorsoplantar flexion RoM (degrees)	93.2 ± 3.5	93.7 ± 3.9	0.64	90.5 ± 3.0	0.37
Inversion‐eversion RoM (degrees)	12.9 ± 7.3	26.9 ± 4.6	**<0.001**	25.6 ± 2.4	**<0.001**
Dorsiplantar flexion^a^					
Initial contact	−5.0 ± 2.7	6.3 ± 1.4	**<0.001**	5.1 ± 1.5	**<0.001**
Loading response	−5.8 ± 0.9	0.5 ± 1.4	**<0.001**	−0.3 ± 1.2	**<0.001**
Mid‐stance	11.2 ± 5.9	17.8 ± 2.5	**<0.001**	14.7 ± 3.9	**0.002**
Terminal stance	−1.2 ± 1.1	−4.5 ± 1.9	**0.003**	−6.7 ± 4.9	**0.001**
Pre‐swing	−13.1 ± 2.6	−8.8 ± 3.9	**0.003**	−10.4 ± 3.9	**0.010**
Swing	4.8 ± 6.2	8.4 ± 4.7	**0.01**	8.8 ± 3.4	**0.010**
Inversion‐Eversion^b^					
Initial contact	13.5 ± 3.8	1.5 ± 3.8	**<0.001**	1.9 ± 5.2	**<0.001**
Loading response	4.4 ± 3.2	−3.4 ± 4.1	**0.003**	−4.1 ± 2.5	**0.002**
Mid‐stance	12.9 ± 7.4	6.3 ± 3.6	**0.004**	4.6 ± 3.3	**0.001**
Terminal stance	11.5 ± 3.2	3.9 ± 3.4	**<0.001**	4.4 ± 5.6	**0.001**
Pre‐swing	−5.9 ± 3.8	−9.6 ± 4.8	**0.003**	−11.3 ± 3.9	**0.001**
Swing	10.8 ± 4.9	4.7 ± 4.1	**0.004**	6.2 ± 5.1	**0.01**

Bold indicates significance values *p* < 0.05.

Abbreviations: RoM, Range of Motion; CAI, chronic lateral ankle instability

a Positive values show plantar flexion and negative values indicate dorsiflexion

^b^ Positive values show inversion and negative values indicate eversion.

Range of motion also showed a significant decrease in the stable ankle of patients with CAI compared to the right (12.9 ± 7.3 *vs* 26.9 ± 4.6; *p* <0.001) and left ankle of the control group (12.9 ± 7.3 *vs* 25.6 ± 2.4; *p* <0.001). Moreover, dorsi‐plantar flexion and inversion‐eversion were significantly altered with a tendency to plantarflexion and inversion in all phases of the gait cycle including initial contact, foot flat, midstance, terminal stance, pre‐swing, and swing (*p* <0.05, Table 4).

## Discussion

### Overview of Study

This study aimed to determine whether unilateral CAI affects the kinematic parameters of the uninjured contralateral ankle. We found that the kinematics of the gait was substantially altered in the uninjured ankles of patients with unilateral CAI. To the best of our knowledge, this is the first report to evaluate the kinematic changes of unilateral CAI on the individual's contralateral uninjured ankle. Our study showed that the uninjured ankle in patients with unilateral CAI had a significant tendency towards plantarflexion and inversion when compared to healthy individuals, showing the same alterations as the unstable side.

### Clinical Application

In a study conducted by Chinn *et al*. comparing kinematics of unstable ankles in 15 patients affected by unilateral CAI with 13 participants as a healthy control population, it was reported that individuals with CAI had less dorsiflexion at 42%–51% of the gait cycle during walking and at 54%–68% of the gait during jogging.^11^ They also reported increased inversion at three points of the gait cycle during jogging at 11%–18%, 33%–39%, and 79%–84% of the gait cycle. Our results highlighted that even the uninjured ankles of patients with unilateral CAI had substantially negative biomechanical alterations when compared to the uninjured control population. The contralateral uninjured ankles in CAI nonetheless demonstrated a significantly greater degree of inversion and a significantly decreased dorsiflexion during the gait cycle than a healthy ankle.

Regarding the RoM of inversion‐eversion, we found that the uninjured ankle of the patients with CAI had lower overall RoM compared to the controls; however, the differences between the controls and the CAI group were not statistically significant regarding the RoM in dorsiplantar flexion. Drewes *et al*. reported a deficit in dorsiflexion of the ankle suffering from CAI and have suggested that this change is attributed to kinematic changes in the talocrural joint among individuals with CAI.^19^ They realized that unstable ankles had less tendency to dorsiflex when compared to the uninjured ankles in healthy individuals and reported that this lower tendency has led to increased risk for a recurrent ankle sprain. Delahunt *et al*. studied both healthy participants and patients with unstable ankles and noted an increased inversion in unstable ankles as well as increased activity of rectus femoris and peroneus longus muscles. They claimed that these changes were protective mechanisms to counteract the increased inversion of the ankle joint.^12^ In the present study, we demonstrated that the uninjured ankles in patients with CAI developed more inversion and less dorsiflexion compared to the healthy controls and compared to the unstable ankle of the patients. Although current treatment guidelines have often recommended initial non‐surgical management of the unstable ankle in unilateral CAI, these data arguably support a more aggressive bilateral rehabilitative protocol that includes an earlier surgical approach to retain stability in the unstable ankle, as delayed treatment or conservative approaches might predispose the normal healthy side to progressive biomechanical deterioration, degenerative changes due to continuous micro‐traumas, and increased risk for similar stability issues and gait derangements.^1^, ^20^, ^21^

Compared to previous literature, the current study performed a more robust assessment of ankle kinematics, including RoM, velocity, and acceleration during gait, as well as an assessment of inversion and eversion during gait. Furthermore, in order to assure consistency between controls, we also compared parameters between the controls and found no statistically significant difference between them.

### Limitations of Study

There were a few limitations that should be taken into consideration. Study participants were asked to walk at their normal speed in their usual state in order to measure velocity and acceleration, which might have caused some bias in the assessment of kinematic variables as some studies have shown that kinematics could vary at different speeds of walking (walking *vs* jogging).^11^ Since exact biomechanical assessment of mechanisms of injury was not possible, differences in injury causation could have caused some heterogeneity among the participants. Another limitation was that due to the low number of participants, we could not perform cluster analysis, multivariate analysis, or correlation analysis to find any relationship between the patients' individual and demographic characteristics and the biomechanical data. Patients' self‐reported outcomes, as well as socioeconomic status, could also have affected the outcomes which were not considered in this study.

### Conclusion

The current study strongly suggests that unilateral chronic ankle instability can affect the kinematics of the contralateral uninjured ankle in patients with unilateral CAI. These changes may include ankle‐toe degree, the velocity of ankle movement, acceleration, and inversion‐eversion RoM. The uninjured ankles of such patients also had a significantly lower tendency towards dorsiflexion during gait when compared to individuals with bilaterally normal ankles. While early and appropriate surgical stabilization for unilateral CAI might be a rational way to prevent this issue, it is recommended that any rehabilitation protocol should include both the unstable and stable ankles.

## Conflicts of Interest

None declared.
